# New Trend in Treating Class II Division 2 Subdivision: A Case Report

**DOI:** 10.7759/cureus.47353

**Published:** 2023-10-19

**Authors:** Ashok Pothuri, Mohanakrishnan PJ, Lingeshkumar N, Mothikrishna N, Vijayadhith Chinnapan

**Affiliations:** 1 Orthodontics and Dentofacial Orthopaedics, Priyadarshini Dental College and Hospital, Tiruvallur, IND

**Keywords:** space closure, dental midline, class i canine, asymmetrical extraction, class ii div 2 subdivision

## Abstract

Class II correction in non-growing patients will always pose a challenge in treatment, especially in a subdivision condition where one quadrant will exhibit class II molar and canine, and the other side will exhibit class I. In such a scenario, a contemporary extraction protocol will face a challenge in symmetric space closure. This case report describes the management of class II subdivision malocclusion by the latest approach, i.e., extraction of a single premolar. At the end of the treatment, midlines were corrected and good functional class I canine relations on both sides were established.

## Introduction

In Angle's class II division 2 subdivision malocclusion, the molar occlusion is class II on one side and class I on the other. Management of class II malocclusion is considered difficult and challenging. Class II subdivision malocclusion can be classified into two types: type 1, characterized by distal positioning of the mandibular first molar on the class II side, and type 2, characterized by mesial positioning of the maxillary first molar on the class II side.

According to Janson et al. [1], the conventional treatment options for type 1 class II subdivision malocclusions depending on the facial profile and/or the amount of crowding will be extractions of two maxillary premolars. This case report describes the management of class II subdivision malocclusion by extraction of a single premolar. Treating the patient in this conservative approach will reduce the extraction of other premolars on the class I molar region. There is very little evidence available to substantiate the following treatment modality. There are a few criteria to fulfill in proceeding with this treatment protocol, which shall be briefly discussed in the case report.

## Case presentation

A male patient aged 17 years came to the Department of Orthodontics and Dentofacial Orthopedics with a chief complaint of crowding in the upper and lower arches.

**Figure 1:**
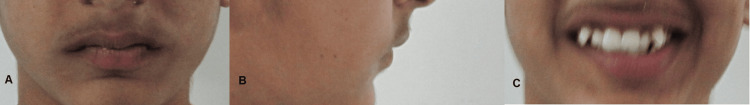
Extraoral photographs A: Frontal view. B: Profile view. C: Frontal smile.

The extraoral examination showed a mild convex profile with an average nasolabial angle and average clinical Frankfort-mandibular plane angle (FMA) (Figure 1).

**Figure 2:**
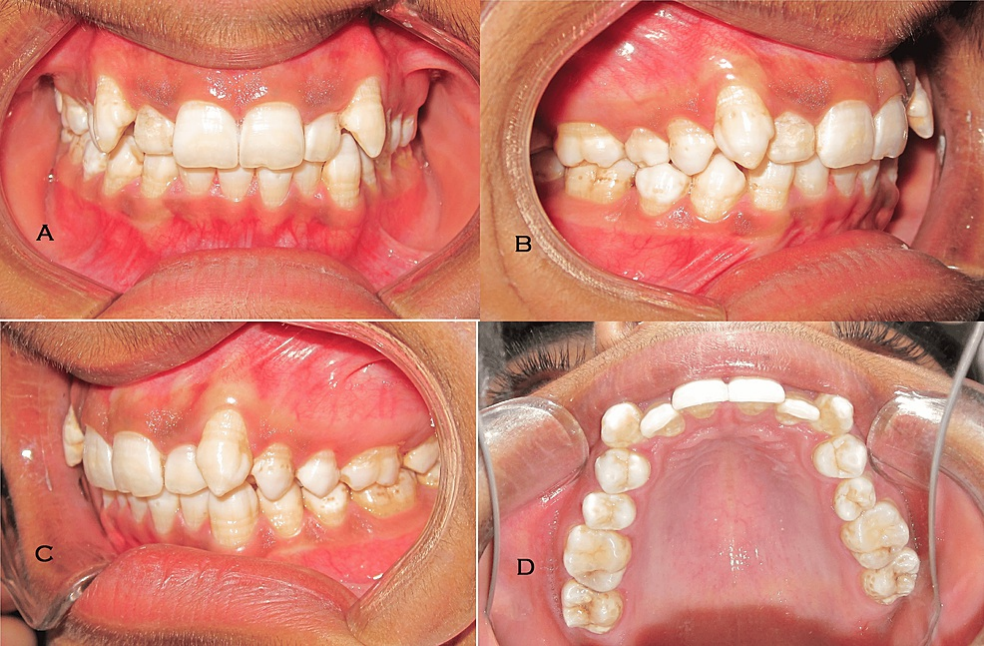
Intraoral photographs A: Frontal. B: Oblique right. C: Oblique left. D: Maxilla occlusal.

**Figure 3:**
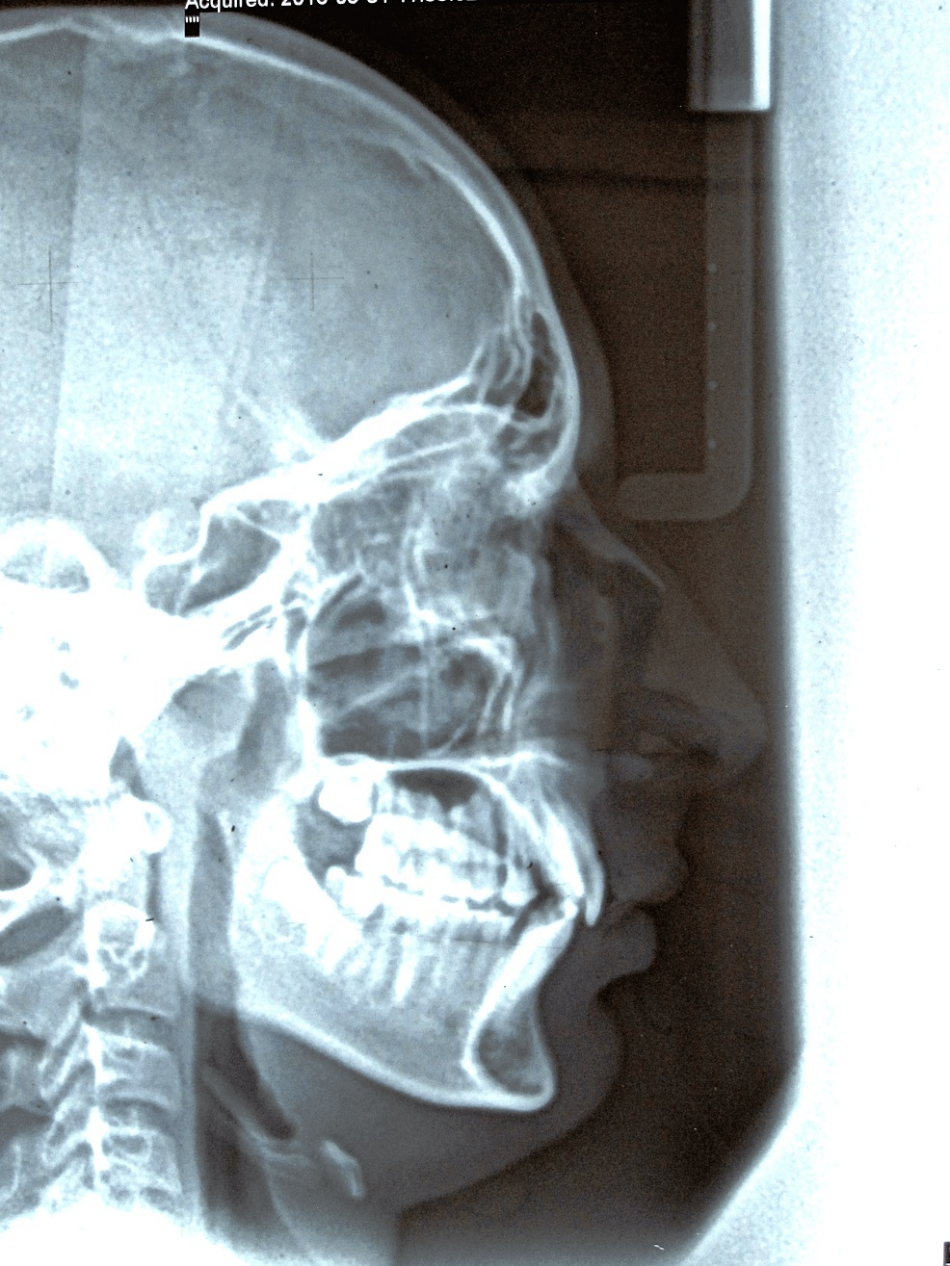
Pretreatment lateral cephalogram

The intraoral examination showed labially placed canines in the upper and mild crowding in the lower with molar relation of Angle’s class I on the right side and class II on the left side. Maxillary midline deviation toward the right side by 4 mm was noted. Generalized fluorosis in the upper and lower arches was present (Figure 2). The cephalometrics showed skeletal class II relation and average growth pattern (Figure 3).

Treatment plan

The treatment plan was designed based on the chief complaint by extraction of a single premolar on the left side for the correction of the class II canine relation and midline and finishing the case in class II on the left and class I on the right side molar region.

Treatment progress

The upper and lower arch first molars were banded along with the transpalatal arch for better anchorage control and bonding was done using 0.022 slot MBT (McLaughlin, Bennett, and Trevisi) fixed appliance after extraction of 24. The initial leveling and alignment were done using 0.014 nickel-titanium (NiTi) wire. It was followed by 0.016 NiTi, 0.016 SS, 0.018 SS, 0.017x0.025 NiTi, 0.017x0.025 SS, and 0.019x0.025 SS. Sliding mechanics with active tiebacks were used to close the extraction space.

**Figure 4:**
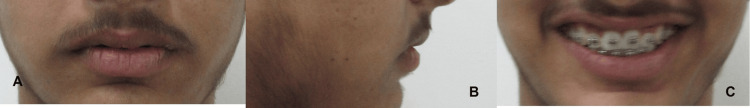
Post-treatment extraoral photographs A: Frontal. B: Profile. C: Frontal smile.

**Figure 5:**
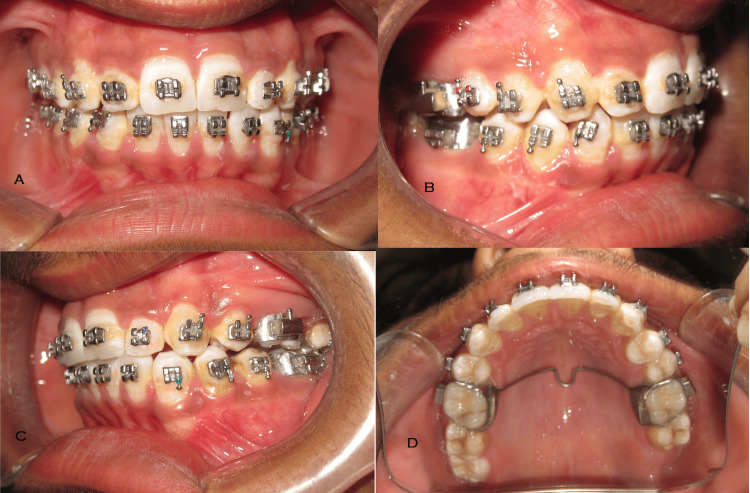
Post-treatment intraoral photographs A: Frontal. B: Oblique right. C: Oblique left. D: Maxilla occlusal.

The finishing and settling were done using 0.014 NiTi wire. Post-treatment results are satisfactory with an ideal facial profile (Figure 4), and intraorally good intercuspation and axial inclination of anterior with normal overjet and overbite. The midlines are matched in upper and lower arches and good canine class I on both sides (Figure 5).

## Discussion

Class II subdivision cases always face a challenge in treatment planning and execution. The following scenario has a few advantages to proceeding with the single premolar extraction, which includes an esthetic profile and favorable midline deviations toward the class II side. If the case exhibited a more convex profile that is most common in class II conditions, a single premolar extraction will worsen the condition. As for the symmetry, the deviation toward class I will provide a positive treatment outcome.

According to Shroff et al. [2], orthodontic correction of dental asymmetries is often considered a difficult and challenging process because of misdiagnosis and poorly planned treatment mechanics. Extraction treatment of class II subdivision may involve the extraction of one, three, or four premolars.

In the present case, extraction of a single premolar (24) was planned followed by fixed appliance therapy (0.022 MBT). MBT appliance was chosen as it is a versatile appliance and uses light forces.

A study done by Janson et al. [3] found that there was no difference in smile attractiveness between the esthetic results of different treatment protocols of class II division 1 subdivision malocclusion, i.e., with one, three, and four premolar extractions. Also, the buccal corridor widths did not differ between the above groups.

Another challenge the present case posed was that of generalized fluorosis which does not allow proper bonding of the brackets. A study done by Opinya and Pameijer [4] recommended 120 and 180 seconds of etching time with 37% phosphoric acid for fluorosed teeth. In this case, etching was done for 120 seconds.

At the end of treatment, the upper and lower arches were well aligned. The upper and lower midline coincided with each other. Post-treatment orthopantomogram showed satisfactory root alignment. Table 1 shows the comparison between pre-treatment and post-treatment cephalometric readings. There is a mild increase in upper and lower incisor inclinations, which is an acceptable pre-treatment profile and the nasolabial angle is obtuse; incidentally, there is no change in the nasolabial angle regardless of the proclined post-treatment incisor position.

**Table 1:** Pre-treatment and post-treatment cephalometric reading

Cephalometric readings	Pre-treatment	Post-treatment
SNA (Sella Nasion Point A)	83 degrees	83 degrees
SNB (Sella Nasion Point B)	76 degrees	78 degrees
ANB (Point A Nasion Point B)	7 degrees	5 degrees
SNGoGn (Sella Nasion Gonion Gnathion)	27 degrees	26 degrees
FMA (Frankfort-mandibular plane angle)	27 degrees	25 degrees
IMPA (Incisor mandibular plane angle)	105 degrees	104 degrees
U1-NA (Angle) (Upper 1 Nasion Point A)	20 degrees	25 degrees
U1-NA (Linear) (Upper 1 Nasion Point A)	2 mm	4 mm
L1-NB (Angle) (Upper Nasion Point B)	30 degrees	33 degrees
L1-NB (Linear) (Upper 1 Nasion Point B)	5 mm	7 mm
Nasolabial angle	115 degrees	115 degrees

Various other alternatives of class II subdivision management are also mentioned in the literature. In their study, Shroff et al. [2] have advocated non-extraction treatment with tip-back moments. Based on studies by Wertz [5] and Burstone [6], they used intermaxillary elastics. For class II correction, Smith and Alexander [7] used cervical pull headgear for the management of class II subdivision with open bite. Other studies by Ross et al. [8] and Bock et al. [9] advocated the use of fixed functional appliances. Likewise, Janson et al. [10] have advocated an orthodontic-surgical approach to manage class II subdivision malocclusion.

## Conclusions

A case of class II division 2 subdivision malocclusion with labially placed upper canines, mild lower anterior crowding, and deviated midline was successfully treated with atypical single premolar extraction. Proper planning and diagnosis will deliver a successful and positive outcome.
